# A Quantifiable Comprehensive Evaluation Method Combining Optical Motion Capture and Simulation—Assessing the Layout Design of Special Vehicle Cabins

**DOI:** 10.3390/s25165053

**Published:** 2025-08-14

**Authors:** Sen Gu, Tianyi Zhang, Hanyu Wang, Qingbin Wang

**Affiliations:** 1 School of Mechanical and Electrical Engineering, Henan University of Technology, Zhengzhou 450001, China; gusen@stu.haut.edu.cn (S.G.); zty_stu@stu.haut.edu.cn (T.Z.); 2 School of Design and Art, Henan University of Technology, Zhengzhou 450001, China; 3 Key Laboratory of Industrial Design and Ergonomics, Ministry of Industry and Information Technology, Northwestern Polytechnical University, Xi’an 710072, China; why5980700why@mail.nwpu.edu.cn; 4 Industrial Design and Ergonomics Innovation Center, Ningbo Institute of Northwestern Polytechnical University, Ningbo 315103, China

**Keywords:** evaluation methodology, man-machine layout optimization, optical motion capture, special vehicles

## Abstract

**Highlights:**

**What are the main findings?**

- A methodology for the ergonomic evaluation of special vehicle cockpits has proposed, which is a quantifiable, comprehensive evaluation method combining optical motion capture and simulation.
- The effectiveness of the evaluation method proposed in this study was validated through an analysis of actual projects.

**What is the implication of the main finding?**

- The resolution of discrepancies between human posture in simulation software and actual operating posture has bee achieved.
- The regionalization and fragmentation of evaluation results in previous simulation software has been improved, and the problem of information silos between evaluation modules has been solved.

**Abstract:**

Ergonomic assessments for specialized vehicle cockpits are often costly, subjective, or fragmented. To address these issues, this study proposes and validates a quantifiable comprehensive evaluation method combining optical motion capture with simulation. The methodology uses motion capture to acquire accurate, dynamic operator posture data, which drives a digital human model in a virtual environment. A novel assessment framework then integrates the results from six ergonomic tools into a single, comprehensive score using a multi-criteria weighting model, overcoming the ‘information silo’ problem of traditional software. In a case study optimizing a flatbed transporter cockpit, the method guided a redesign that significantly improved the overall ergonomic score from 0.422 to 0.277. The effectiveness of the optimization was validated by a 40% increase in key control accessibility and a significant reduction in electromyography (EMG) signals in the neck, shoulder, and lumbar regions. This study provides an innovative, data-driven methodology for the objective design and evaluation of customized human–machine systems, demonstrating its utility in reducing physical strain and enhancing operator comfort, with broad applicability to other complex industrial environments.

## 1. Introduction

In the context of accelerated urbanization and economic development, specialized vehicles are assuming an increasingly significant role in pivotal domains such as logistics and infrastructure [1]. These vehicles are typically highly customized for specific tasks, resulting in significant differences in cabin size and layout between different batches [2]. Consequently, the human–machine interface of each batch must be designed independently. The configuration of the cabin’s human–machine layout is a pivotal element that exerts a direct influence on the driver’s operating comfort, work efficiency, and even driving safety.

In order to optimize cockpit layouts, various evaluation methods have been explored in academia. Conventional experimental methodologies employ physiological data collection techniques, such as electromyography (EMG) [3] and pressure distribution [4], to ascertain the impact of layout on the driver’s physiological load. Nevertheless, this approach is encumbered by the necessity for physical prototypes and costly physiological signal acquisition apparatus, rendering it impractical for the customized production of specialized vehicles, which commonly entails multiple batches and limited production runs. In order to circumvent the substantial expenses associated with experimental methods, researchers have increasingly resorted to mathematical models [5,6,7] and algorithms [8,9,10,11] for optimization purposes. Notable examples of such algorithms include particle swarm and genetic algorithms. Nevertheless, it should be noted that these methods predominantly offer a single perspective in their modelling (e.g., joint comfort), and the solutions they yield frequently manifest as local optima rather than global optima. Furthermore, highly theoretical models are subject to considerable limitations in terms of their practical application when confronted with complex engineering and manufacturing constraints. In light of these developments, human factor engineering simulation software programs such as JACK and CATIA have emerged as the prevailing assessment tools, owing to their merits of eliminating the necessity for physical prototypes and facilitating expeditious iteration [12,13,14,15,16]. Such software integrates multiple assessment modules, including accessibility, field of view, and comfort. Theoretically, such software is capable of performing a comprehensive analysis [17,18,19,20,21]. However, in practical applications, two fundamental research gaps have been identified: (1) Accuracy of simulation posture: Conventional simulation methods depend on the utilization of predefined posture libraries incorporated within the software, or on manual adjustments, which bear a substantial discrepancy to the actual postures adopted by drivers in real, dynamic operational scenarios. This has the effect of undermining the accuracy and reliability of assessment results. (2) Fragmentation of assessment results and integration challenges: It has been established that multiple assessment modules within the software (e.g., RULA, OWAS, LBA) operate independently, thereby producing multiple discrete and non-standardized results for the same layout scheme. This has the effect of creating ‘information silos’. Designers are unable to attain a unified, quantifiable, comprehensive score with which to objectively evaluate the layout quality. This remains a cause of assessment processes that are heavily reliant on subjective experience.

In order to address the aforementioned research gaps, the present study aims to address the following core scientific questions: Question 1: Which methodology can be employed to accurately capture the dynamic posture of the human body under real-world conditions and utilize it as a simulation input to ensure the accuracy of the assessment data at its source? Question 2: Which methodology can be employed to integrate the multifarious, disparate assessment results from simulation software in order to establish a scientific, quantitative, and comprehensive evaluation model for the objective optimization of cockpit layouts?

In response to the aforementioned issues, the present study aims to propose and validate a quantitative comprehensive evaluation method that combines optical motion capture and simulation. The integration of high-precision posture data and a multi-module evaluation system is a key feature of this method, which aims to overcome the limitations of existing methods. The primary contributions of this study are as follows:

- A high-fidelity posture data acquisition and import method is proposed, integrating optical motion capture technology. This ensures a high degree of consistency between the posture of the simulated digital human model and the actual working posture, thereby solving the problem of source accuracy in simulation assessment.
- A quantitative and comprehensive integrated assessment framework is constructed. The framework under discussion integrates six distinct assessment methods, including a field of view analysis, reachability analysis, RULA, and OWAS. The integration of data quantification and normalization, in conjunction with the implementation of the CRITIC weighting method, facilitates the consolidation of disparate assessment outcomes into a unified, comprehensive score. This approach addresses the issue of ‘information silos’ between assessment modules, thereby enhancing the objectivity and practicality of the assessment process.
- The efficacy of the method has been substantiated through empirical real-world case studies. To illustrate this point, consider the optimization project of the cab of a specific type of flatbed transport vehicle. The practical application effectiveness of this method has been validated, demonstrating that it can effectively lead to improved operational comfort and reduce fatigue risks.

The present paper is comprised of five chapters. The initial chapter delineates the research background and significance of the present study. In Section 2, the reader is introduced to the specific steps of the optimization design method, as well as the composition modules, evaluation rules, and evaluation indicators of the assessment method. Section 3 employs the optimization design method in specific practical scenarios to validate its feasibility. In the discussion section of Section 4, a cross-validation of the optimization results is conducted using a simulation analysis and muscle electrical signals. This is done in order to analyze the feasibility and limitations of the optimization design method. Section 5 summarizes the research content and provides a framework for future directions.

## 2. Materials and Methods

**Figure 1:**
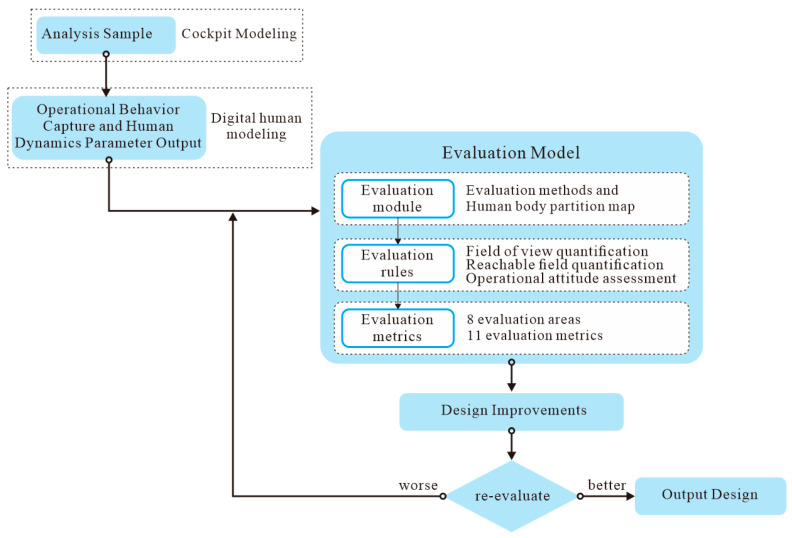
Design method for human–machine layout in special vehicle cabins.

In this study, a mixed motion capture and simulation method is proposed for the quantifiable and comprehensive evaluation of special vehicle cab layouts. This method employs optical sensors to obtain posture feature information of operators inside the special vehicle cab, and utilizes a multi-method, multi-data fusion evaluation model to comprehensively evaluate the human–machine layout of the special vehicle cab. This approach resolves the issue of information silos between different evaluation methods. The implementation of this comprehensive evaluation method is achieved through the following four steps, with the overall process illustrated in Figure 1.

The initial step in the process is the capture of operational behavior and the subsequent output of human kinematic parameters. The utilization of an optical 3D motion capture system facilitates the acquisition of human kinematic data, encompassing 3D coordinates, Euler angles, and quaternions, pertaining to the operator’s posture during operation. Subsequent to this, the obtained data are integrated into simulation software, thereby facilitating the generation of a digital human motion posture model.

The subsequent step is the determination of the evaluation module. The integration of existing single evaluation methods has resulted in the construction of a multi-method fusion evaluation, with human body partition mapping being utilized to conduct targeted analyses of local and overall postures.

The third step in the process is the establishment of evaluation rules. The creation of a comprehensive evaluation model was achieved by integrating the three evaluation indicators, including a comprehensive field of view evaluation, operational accessibility evaluation, and operational posture rationality evaluation, with human body zone mapping. The model encompasses eight evaluation areas and 11 evaluation methods.

The fourth step in the process is the calculation of evaluation indicators. The evaluation of operating posture is derived from the synthesis of quantitative field of view and field of reach assessments, amalgamated with supplementary evaluation methodologies. The evaluation results are then normalized, and the weights of each evaluation method are calculated.

### 2.1. Operational Behavior Capture and Human Dynamics Parameter Output

The present study employed an optical 3D motion capture system (NOKOV Science & Technology Co., Ltd., Beijing, China) for the purpose of posture recognition, with the concomitant collection of relevant human kinematic data. The optical 3D motion capture system has been demonstrated to possess the capability to accurately and comprehensively track human node information. In comparison with alternative positioning methodologies, such as inertial motion capture and GPS positioning systems, optical lens motion capture possesses the advantageous qualities of high accuracy, low latency, and robust real-time performance.

**Figure 2:**
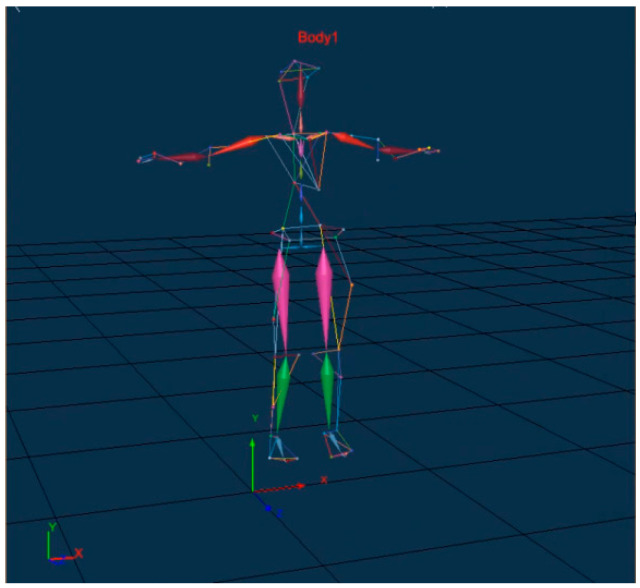
The human digital skeleton formed by node connections.

Optical motion capture cameras were utilized to track the nodes of a stationary human body, and an identification program was constructed based on XINGYING (Version 3.3.1) to generate the corresponding human skeleton diagram, as illustrated in Figure 2.

**Figure 3:**
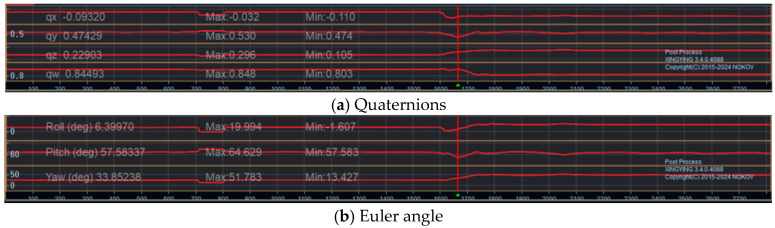
Quaternions and Euler angles of the human body in task posture.

Secondly, the experimenter provides the operator with task instructions. The kinematic parameters of the human body in the working posture are collected through node tracking by the optical 3D motion capture system. The utilization of a recognition programme facilitates the extraction of the quaternions and Euler angles corresponding to the task posture, as illustrated in Figure 3.

**Figure 4:**
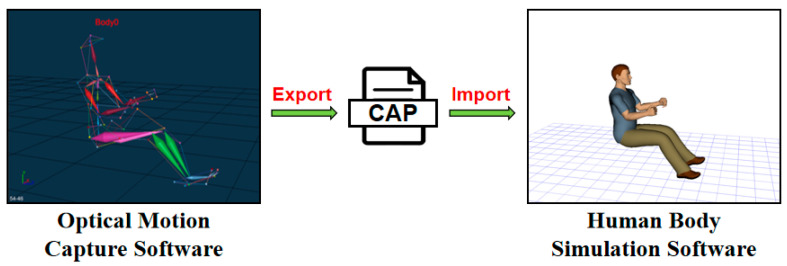
Data conversion process.

The human kinematic parameters generated via software should be subsequently exported, followed by a thorough examination of the biomechanical validity of the exported file. It is imperative to ensure that the joint bending angles remain within physiological limits. Following the completion of data verification and the inspection of the coding and syntax, the posture obtained from the optical motion capture can be imported into human simulation software to obtain the corresponding human model, as illustrated in Figure 4.

### 2.2. Evaluation Module

The present study developed a multi-method fusion evaluation approach, building upon the existing single-method evaluation framework. The objective of the present study was to comprehensively assess human–machine interaction tasks and human posture in the design of special vehicle cab layouts, with a view to evaluating and optimizing such layouts. The single evaluation methods employed in this study included a visual analysis (VA), reachability analysis (RA), rapid upper limb assessment (RULA), lower back analysis (LBA), Ovako Working Posture Analysis System (OWAS), and comfort assessment (CA).

#### 2.2.1. Visual Analysis

The VA defines the total area that an operator can see by simply turning their head while performing tasks in the cockpit. The primary function of the system is to analyze the operator’s field of vision, blind spots, and color recognition ability within the cockpit. The purpose of this analysis is to reduce eye movement and adjustments during work, thereby ensuring good visibility for the operator. This, in turn, serves to lower the risk of accidents, reduce driving fatigue and visual stress, and effectively improve their work efficiency.

Assuming that the human eye is located at the origin $O\left(0,0,0\right)$ of the coordinate system, with the line of sight along the positive z-axis direction, the visual cone consists of six planes: the near clipping plane, the far clipping plane, the left plane, the right plane, the upper plane, and the lower plane.

The equation for the near clipping plane, which defines the closest boundary of vision, is:

(1) z=n,

Within the equation, z represents the variable z-coordinate of any point in three-dimensional space, while the constant *n* signifies the near clipping distance, which represents the distance from the nearest visible boundary of the frustum to the human eye. In this study, n is set to 0.3 m.

Similarly, the equation for the far clipping plane, which defines the furthest boundary of vision, is:

(2) z=f,

In the equation, the constant f is the far clipping distance, which represents the distance from the farthest visible boundary of the frustum to the camera. In this study, the driver’s cab space is relatively small; consequently, f is set to 2 m.

Given the symmetrical nature of the horizontal viewing angle, the left and right planes are determined by half of the horizontal viewing angle. The formulas for the left and right boundary planes are as follows:

(3) $$x=\;\pm \;z\cdot \tan\left(\frac{\theta_{h}}{2}\right),$$

Within the formula, x denotes the coordinate of point x and $\theta_{h}$ signifies the horizontal field of view of the human eye, which is conventionally taken to be 120° in a driving environment.

The upper and lower boundary plane formulas are:

(4) $$y=\;\pm \;z\cdot \tan\left(\frac{\theta_{v}}{2}\right),$$

Within the formula, y denotes the coordinate of point y, $\theta_{v}$ signifies the vertical field of view of the human eye, and the value is 60° when calculating the upper boundary and 70° when calculating the lower boundary.

In summary, the spatial range of the cone of vision can be expressed as follows:

(5) $$\left\{\begin{array}{c} -z\cdot \tan\left(\frac{\theta_{h}}{2}\right)\leq x\leq z\cdot \tan\left(\frac{\theta_{h}}{2}\right)\\ -z\cdot \tan\left(\frac{\theta_{v}}{2}\right)\leq y\leq z\cdot \tan\left(\frac{\theta_{v}}{2}\right)\\ n\leq z\leq f \end{array}\right.,$$

The field of view is defined as the range of the visual cone covered by head rotation, which is achieved through the cervical spine. This concept encompasses three main degrees of freedom: the yaw angle (horizontal rotation), pitch angle (vertical rotation), and roll angle (head tilt). The present study focuses primarily on the horizontal and vertical ranges of rotation. The spatial range of the field of view can be expressed by the following formula:

(6) $$\left\{\begin{array}{c} -z\cdot \tan\left(\frac{\theta_{h}+\theta_{Yaw}}{2}\right)\leq x\leq z\cdot \tan\left(\frac{\theta_{h}+\theta_{Yaw}}{2}\right)\\ -z\cdot \tan\left(\frac{\theta_{v}+\theta_{Pitch}}{2}\right)\leq y\leq z\cdot \tan\left(\frac{\theta_{v}+\theta_{Pitch}}{2}\right)\\ n\leq z\leq f \end{array}\right.,$$

In the formula, $\theta_{Yaw}$ is the horizontal rotation angle of the head and $\theta_{Pitch}$ is the vertical pitch angle of the head. The range of values is displayed in Table 1.

A visual obstruction is defined as a situation in which the driver’s field of vision is impeded by objects such as the steering wheel or equipment within their visual range. In contradistinction to blind spots, visual obstructions are localized blockages within the field of vision and are typically analyzed within the driver’s cabin. The parametric equation of the straight line from viewpoint O(0,0,0) to target point $P(x_{p},y_{p},z_{p})$ is as follows:

(7) $$\vec{r}\left(t\right)=t\cdot \vec{P}=\left(tx_{p},{ty}_{p},tz_{p}\right),\;\;t∋\left[0,1\right],$$

The intersection point with the obstacle plane is defined by the following parameters:

(8) $$t_{0}=\frac{\vec{n}\cdot \vec{O}S}{\vec{n}\cdot \vec{O}P},$$

The center coordinates of the obstacle are denoted by $S(x_{s},y_{s},z_{s})$, the normal vector of the obstacle by $\vec{n}=(x_{n},y_{n},z_{n})$, and the intersection coordinates by:

(9) $$Q=\left(t_{0}x_{p},{t_{0}y}_{p},t_{0}z_{p}\right),$$

It is imperative that an obstruction fulfils all three of the following criteria:

(1) $0<t_{0}<1$;
(2) the intersection point Q is within the obstacle geometry;
(3) Q is within the field of view.

#### 2.2.2. Reachability Analysis

The concept of reachability can be categorized into upper limb reachability and lower limb reachability, based on the specific limbs involved. The classification of the object under discussion can be further refined according to the principle of comfortable reachability and maximum reachability.

The upper limb’s reachability is determined by the creation of an arm dynamics model with a fixed limb length and joint mobility. This is followed by the analysis of the spatial range that the human body can reach in specific tasks. Subsequently, the model accurately simulates the boundaries of human movement. This facilitates the rapid identification of design flaws and the formulation of improvement plans. The upper limb comfort reachability formula is derived from a shoulder–elbow chain structure model:

(10) $$\left\{\begin{array}{l} x=L_{1}\cos\theta_{1}\cos\phi_{1}+L_{2}\cos\left(\theta_{1}+\theta_{2}\right)\cos\left(\phi_{1}+\phi_{2}\right)\\ y=L_{1}\sin\theta_{1}+L_{2}\sin\left(\theta_{1}+\theta_{2}\right)\\ z=L_{1}\cos\theta_{1}\sin\phi_{1}+L_{2}\cos\left(\theta_{1}+\theta_{2}\right)\sin\left(\phi_{1}+\phi_{2}\right) \end{array}\right.,$$

In the formula, $L_{1}$ represents the upper arm length, $L_{2}$ represents the lower arm length, $\theta_{1}$ is the flexion or extension angle of the shoulder joint in the sagittal plane, $\phi_{1}$ is the abduction or adduction angle of the shoulder joint in the coronal plane, $\theta_{2}$ is the flexion or extension angle of the elbow joint in the sagittal plane, and $\phi_{2}$ is the pronation or supination angle of the forearm in the horizontal plane. The range of angle values is demonstrated in Table 2.

It has been demonstrated that trunk movement causes displacement of the shoulder joint origin, and maximum reachability is based on the shoulder–elbow chain structure model with the introduction of trunk displacement parameters. The expression for the distance of the shoulder origin forward displacement caused by trunk forward flexion is as follows:

(11) $$\Delta x=L_{t}\sin\theta_{3},$$

In the formula, Δx is the distance of the shoulder origin forward displacement, $L_{t}$ is the trunk length, and $\theta_{3}$ is the trunk flexion angle.

The expression for the distance of shoulder origin forward displacement caused by trunk lateral curvature is as follows:

(12) $$\Delta y=L_{t}\sin\phi_{3},$$

In the formula, Δy is defined as the lateral displacement distance of the shoulder origin, and $\phi_{3}$ is specified as the lateral bending angle of the trunk. The range of values is demonstrated in Table 3.

It is evident that based on the principle of trunk displacement, the superimposition of the arm movement model can yield the maximum reachability range of shoulder–elbow–waist coordination. This can be expressed as follows:

(13) $$\left\{\begin{array}{l} x=\Delta x+L_{1}\cos\theta_{1}\cos\phi_{1}+L_{2}\cos\left(\theta_{1}+\theta_{2}\right)\cos\left(\phi_{1}+\phi_{2}\right)\\ y=\Delta y+L_{1}\sin\theta_{1}+L_{2}\sin\left(\theta_{1}+\theta_{2}\right)\\ z={L_{t}\cos\theta_{3}\cos\phi_{3}+L}_{1}\cos\theta_{1}\sin\phi_{1}+L_{2}\cos\left(\theta_{1}+\theta_{2}\right)\sin\left(\phi_{1}+\phi_{2}\right) \end{array}\right.,$$

The reachability of the lower limbs is divided into two categories: the comfortable reachability of the foot driven by the knee joint and the maximum reachability of the foot driven by the knee–hip joint. The foot end coordinates $\left(x_{f},y_{f}\right)$ are determined in conjunction with the hip joint and knee joint angles, and the foot position formula is as follows:

(14) $$\left\{\begin{array}{c} x_{f}=L_{thigh}\sin\theta_{h}-L_{shank}\sin\left(\theta_{h}+\theta_{k}\right)\\ y_{f}=L_{thigh}\cos\theta_{h}-L_{shank}\cos\left(\theta_{h}+\theta_{k}\right) \end{array}\right.,$$

In the formula, $L_{thigh}$ denotes the length of the thigh, $L_{shank}$ signifies the length of the lower leg, $\theta_{h}$ represents the hip joint angle, and $\theta_{k}$ designates the knee joint angle.

In the event of only the knee joint being driven, the hip joint is fixed, i.e., $\theta_{h}=\theta_{h0}$, and only the knee joint angle $\theta_{k}$ changes. At this particular juncture, the center coordinates $\left(A,B\right)=(L_{thigh}\sin\theta_{h0},L_{thigh}\cos\theta_{h0})$ and the radius $R=L_{shank}$. The arc range is defined as $\theta_{k}\in \left[\theta_{k\_min},\;\theta_{k\_max}\right]$ and the parametric equations are as follows:

(15) $${\left(x_{f}-A\right)}^{2}+{\left(y_{f}-B\right)}^{2}={L^{2}}_{shank},$$

When the knee joint and hip joint are driven in unison, the reachability boundary is composed of four parameterized curves, as illustrated in Table 4.

#### 2.2.3. Rapid Upper Limb Assessment

The RULA is principally utilized to evaluate the impact of upper limb postures and movements (e.g., the neck, shoulders, arms, wrists) in the workplace on the human musculoskeletal system, with a particular emphasis on repetitive movements or static postures. The RULA posture evaluation form (Table 5) is the core tool for scoring, converting the posture angles, loads, and movement frequencies of various body parts into scores to ultimately determine the risk level.

The posture score can be calculated using the following formula:

(16) $$P_{T}=P_{A}+P_{B}+\Delta w,$$

In the formula, $P_{T}$ denotes the posture score, $P_{A}$ represents the posture score for group A, $P_{B}$ signifies the posture score for group B, and Δw is the wrist rotation score, which can take a value of either 0 or 1.

The load and muscle use also affect the final posture score. See Table 6 for the scoring table.

The total score should then be adjusted according to the load score and muscle usage score, as outlined in Table 7.

The total score can then be calculated using Formula (17):

(17) $$P_{RULA}=P_{T}+\Delta e,$$

In the formula, $P_{RULA}$ is the overall score of the RULA tool and Δe is the additional score in Table 7.

RULA assessment scores (1–7 points) are categorized into four risk levels, with higher scores denoting increased risk: 1–2 points indicates low risk, 3–4 points indicates medium risk, 5–6 points indicates high risk, and 7 points indicates extremely high risk. The scoring and level correspondence table can be found in Table 8.

#### 2.2.4. Lower Back Analysis

The LBA is a method that uses biomechanical models to quantify the forces acting on the spine (particularly the lumbar spine) during specific tasks. The tool is frequently utilized for the purpose of evaluating the prospective risk of injury to the lumbar spine, attributable to occupational postures or lifting maneuvers. The fundamental principle underpinning this approach entails the calculation of compressive force, shear force, and torque on the lumbar spine through the utilization of mechanical formulas. A subsequent step involves the comparison of these values with safety thresholds, followed by a comprehensive analysis of the impact of forces on the human spine within specific environmental contexts. It has been demonstrated that an increase in pressure results in a corresponding increase in discomfort in posture. Lumbar torque is defined as the torsional effect on the lumbar spine caused by external loads and body weight, and the formula used to calculate this is as follows:

(18) $$M_{L5/S1}=F_{load}\times d_{load}+F_{body}\times d_{body},$$

In the formula, $F_{load}$ denotes the weight of the external load, $d_{load}$ signifies the horizontal distance from the load’s center of gravity to the lumbar vertebrae L5/S1, $F_{body}$ represents the weight of the human body, and $d_{body}$ indicates the horizontal distance from the center of gravity of the upper body to the lumbar vertebrae L5/S1.

The lumbar compression force is defined as the pressure exerted vertically on the intervertebral disc, primarily generated by muscle force and external loads. The formula is as follows:

(19) $$F_{comp}=\frac{M_{L5/S1}}{d_{muscle}}+F_{vertical}=\frac{M_{L5/S1}}{d_{muscle}}+F_{load}+aF_{body},$$

Within the formula, $d_{muscle}$ denotes the lever arm of the spinal extensor muscles, with a typical range of 5–7 cm. $F_{vertical}$ represents the total force in the vertical direction, which is generally equivalent to the external load in addition to the body’s weight. The coefficient of self-weight for the upper body, a, typically varies from 40% to 60%.

Lumbar shear force is defined as a transverse force that is parallel to the intervertebral disc plane, and is related to the angle of forward lean of the body. The formula is as follows:

(20) $$F_{shear}=F_{load}\sin\theta_{3}+F_{body}\sin\theta_{3},$$

In the formula, $\theta_{3}$ is the trunk forward tilt angle.

#### 2.2.5. Ovako Working Posture Analysis System

The OWAS assesses and codes work postures of the back, arms, and lower limbs, as well as work postures when carrying loads. The classification of work posture risks, as determined by its coding system, is divided into four levels, designated from 1 to 4, with levels 1 and 2 categorized as low-risk and levels 3 and 4 categorized as high-risk. Each level is associated with distinct posture ratings and correction requirements. The posture rating rules are delineated in Table 9.

The classification of posture combinations into four risk levels is achieved by observing and recording the posture codes of the aforementioned four areas and comparing them with the standardized OWAS table, as demonstrated in Table 10.

#### 2.2.6. Comfort Assessment

The CA primarily analyzes the range of motion of specific joints in a human model under designated postures, and selects different comfort reference data for analysis based on the research focus. As is customary in academic discourse, the following comfort reference data are frequently cited: Porter, Krist, Grandjean, and Rebiffe. The present study employs the Krist database as the principal instrument for the CA evaluation. Whilst the majority of databases concentrate on comfort reference data for individual joints, the Krist study examines comfort across multiple joints in overall postures, encompassing multiple body regions including the neck, shoulders, back, hips, left and right arms, and left and right legs.

The Krist database is a comprehensive evaluation tool that analyzes the comfort of work postures from three distinct perspectives: joint angles, posture scores, and biomechanical analyses. These evaluations are informed by prior experimental data and ergonomic models. The comfortable range of joint angles is typically based on the anatomical neutral position, and the quantification formula is as follows:

(21) $$D=\left|\theta_{ture}-\theta_{neutral}\right|,$$

In the formula, D is the deviation angle, $\theta_{ture}$ is the actual joint angle, and $\theta_{neutral}$ is the neutral position angle. When the deviation angle exceeds a certain value, the posture is designated as high-risk. The range of values for the joint variable is presented in Table 11.

The posture score is calculated based on the total score of joint deviation, using the following formula:

(22) $$P=\sum_{i=1}^{n}w_{i}\cdot D_{i},$$

In the formula, P is the posture score, $w_{i}$ is the weight coefficient of each joint, and $D_{i}$ is the deviation grade. The values of $D_{i}$ are displayed in Table 12.

#### 2.2.7. Mapping of Assessment Modules to Human Regions

**Figure 5:**
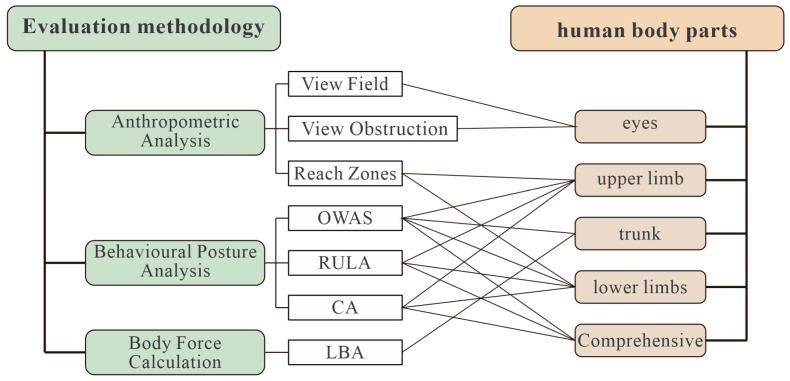
Evaluating the relationship between tool and body part adaptation.

The applicability of existing assessment methods to different fields is due to their varying degrees of relevance. The human body is divided into specific regions, and corresponding assessment methods are applied to each region. This enables targeted analyses of local and overall posture (see Figure 5). The classification criteria primarily depend on two factors. Firstly, it is necessary to ascertain whether the target body part can be rated. Secondly, it is important to determine whether the assessment scope covers the entire body posture or is limited to local limbs. The classification of existing single assessment methods based on the specific characteristics of their assessment objects is the foundation for the subsequent construction of a systematic posture comfort assessment framework.

### 2.3. Evaluation Rules

**Figure 6:**
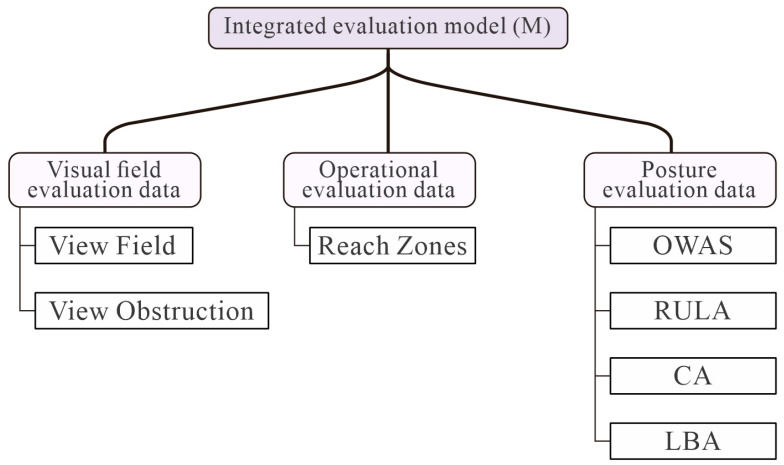
Evaluate input data types and corresponding individual evaluation methods.

In order to integrate data from various assessment modules, it is necessary to quantify non-quantitative data. The present study employs a cumulative scoring system to comprehensively evaluate the current layout, with higher scores indicating poorer comprehensive indicators for the current layout. This evaluation is based on the quantification and normalization of data from various assessment modules. The data sources for the assessment modules are illustrated in Figure 6.

Visual field assessment data comprise two key elements: the visual field range and visual field obstruction. A visual field range analysis adheres to the following principles. In the generation of a hemispherical conical visual field, the ratio of the number of controls outside the visual field range to the total number of controls must be calculated. In the evaluation of color recognition controls, the ratio of the number of controls that can recognize the corresponding color to the total number of controls of that color is to be utilized as the score. Furthermore, the obstruction range of large in-vehicle operating devices, such as the steering wheel and instruments within the cockpit during the working posture, must be calculated. The ratio of the number of controls within the obstruction range to the total number of controls must then be used as the final score.

The following set of rules governs the reachability assessment data. The controls inside the cockpit are divided into three types. Controls that are operated more than or equal to four times per minute are considered intensive controls. Intensive controls should be within the comfortable reachability range of the upper limbs. The ratio of the number of intensive controls outside the comfortable reachability range of the upper limbs to the total number of intensive controls is used as the score for the comfortable reachability range of the upper limbs. Controls that are operated less than four times per minute are considered secondary controls. Secondary controls should be within the reachability range of double-joint (shoulder–waist or knee–hip) movements. The ratio of the number of secondary controls located outside the reachability range of double-joint movements to the total number of secondary controls is utilized as the score for the reachability range of double-joint movements.

Operational posture assessment data are categorized using the RULA, LBA, OWAS, and CA methods. In this study, the OWAS and RULA are employed as tools to assess the cumulative score of the subject. The utilization of these tools does not necessitate quantitative processing; rather, they require only normalization in order to be incorporated into the total score. LBA data are calculated by a biomechanical model based on the current posture, with a value range of 0–6000 N. For the CA comfort assessment, the Krist database was selected for an analysis. The database provides a comfort range of 0–80, with lower scores indicating higher comfort. Following the process of normalization, the result is integrated into the overall assessment score.

### 2.4. Evaluation Metric

This study proposes an evaluation framework for the assessment of cockpit layouts in special-purpose vehicles, with a focus on operational comfort. The proposed criteria encompass a comprehensive field-of-view assessment, an assessment of operational accessibility, and an evaluation of the rationality of operational postures. These criteria are derived from an analysis of the types of human–machine interaction tasks observed in the cockpits of special-purpose vehicles. In order to evaluate these criteria with greater precision, the three indicators are mapped to the following domains for an analysis by incorporating the concept of human body zones: a field of view analysis, field of view obstruction analysis, upper limb reachability analysis, lower limb reachability analysis, upper limb posture rationality analysis, lower limb posture rationality analysis, trunk posture rationality analysis, and comprehensive posture rationality analysis. A range of evaluation methods were employed in order to facilitate a more comprehensive analysis of the advantages and disadvantages of the space layout of special vehicle cockpits.

**Figure 7:**
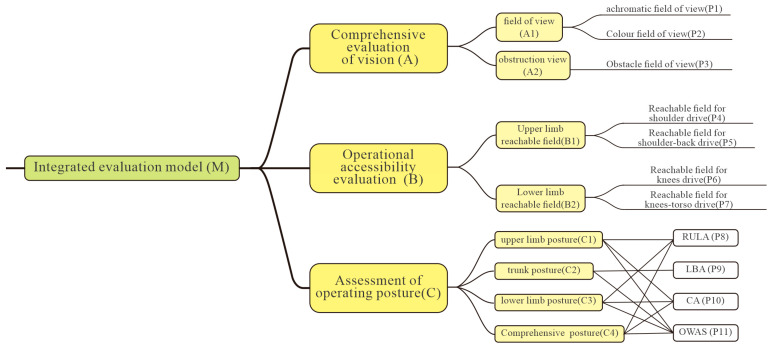
Special vehicle cockpit human–machine layout evaluation model.

The first step in the process is to conduct a comprehensive evaluation of the cockpit layouts of special-purpose vehicles. This evaluation is based on 8 assessment areas and 11 evaluation methods. The relationship between the assessment indicators and evaluation methods is illustrated in Figure 7.

The assessment model is divided into three modules. Module A is a comprehensive field of vision assessment, including field of vision range assessment indicator A1 and field of vision obstruction assessment indicator A2. Module B is an operational reachability assessment, incorporating upper limb reachability assessment indicator B1 and lower limb reachability assessment indicator B2. Module C is the operational posture assessment, which includes upper limb posture rationality assessment indicator C1, lower limb posture rationality assessment indicator C2, trunk posture rationality assessment indicator C3, and comprehensive posture rationality assessment indicator C4. Specifically, within the three-level structure of the assessment model, P1 corresponds to the colorless recognition range assessment method, P2 is the colored recognition range assessment method, P3 is the field of vision obstruction range assessment method, P4 is the shoulder joint drive reachability assessment method, and P5 is the shoulder–waist coordinated drive reachability assessment method. P6 is the knee joint reachability assessment method, P7 is the knee–hip coordinated reachability assessment method, P8 is the RULA rapid upper limb posture assessment method, P9 is the LBA lower back stress analysis assessment method, P10 is the CA comfort assessment method, and P11 is the OWAS work posture assessment method.

The second step in the process involves the quantification of non-quantitative data (P1–P7), as illustrated in Equation (23).

(23) $$P_{1-7}=\frac{x_{t}-x_{a}}{x_{t}},$$

In the formula, $x_{t}$ denotes the total number of corresponding controls under each evaluation condition, and $x_{a}$ represents the number of corresponding controls within the evaluation scope under each evaluation condition.

The third step of the process involves the normalization of the quantified data (P8–P11), as demonstrated in Equation (24).

(24) $$P_{8-11}=\frac{x_{b}-x_{min}}{x_{max}-x_{min}},$$

In the formula, $x_{max}$ represents the maximum value range for each evaluation condition, $x_{min}$ represents the minimum value range for each evaluation condition, and $x_{b}$ represents the score for the current value under each evaluation condition.

The fourth step of the process involves the application of the mean method to calculate the mean of the different evaluation data under each layout evaluation domain. This is illustrated in Table 13.

In the formula, $M_{\left(A1\right)}$ denotes the field of vision assessment score, $M_{\left(A2\right)}$ is the field of vision obstruction assessment score, $M_{\left(B1\right)}$ is the upper limb reachability assessment score, and $M_{\left(B2\right)}$ is the lower limb reachability assessment score. $M_{\left(C1\right)}$ denotes the upper limb posture rationality assessment score, $M_{\left(C2\right)}$ is the lower limb posture rationality assessment score, $M_{\left(C3\right)}$ is the trunk posture rationality assessment score, and $M_{\left(C4\right)}$ is the comprehensive posture rationality assessment score.

It is important to note that the RULA, CA, and OWAS present different assessment data in different operational posture rationality assessments. In the calculation of the layout assessment scores of $M_{\left(C1\right)}$, $M_{\left(C2\right)}$, $M_{\left(C3\right)}$, and $M_{\left(C4\right)}$, it is evident that $P_{8}$, $P_{10}$, and $P_{11}$ exhibit divergent assessment scores across various analysis domains.

In the fifth step of the process, the CRITIC weighting method is employed to calculate the weight values for each evaluation area. The calculation is as follows:

① Data standardization: Since the scores for each assessment area have already been standardized, no additional processing is required.
② Calculate the contrast intensity (standard deviation): Calculate the contrast intensity for each assessment area using Formula (25).

(25) $$\sigma_{j}=\sqrt{\frac{1}{n}\sum_{i=1}^{n}{\left({x’}_{ij}-{\bar{x}’}_{j}\right)}^{2}},$$

In the formula, ${x’}_{ij}$ denotes the value of the i-th sample in the j-th evaluation domain, ${\bar{x}’}_{j}$ is the mean value of the j-th evaluation domain, and n is the number of samples.

③ Calculate the correlation coefficient matrix: The calculation of the Pearson correlation coefficient (Formula (26)) is required to determine the correlation between each assessment area, with the subsequent generation of a correlation coefficient matrix.

(26) $$r_{jk}=\frac{\sum_{i=1}^{n}\left({x’}_{ij}-{\bar{x}’}_{j}\right)\left({x’}_{ik}-{\bar{x}’}_{k}\right)}{\sqrt{{\sum_{i=1}^{n}\left({x’}_{ij}-{\bar{x}’}_{j}\right)}^{2}}\sqrt{{\sum_{i=1}^{n}\left({x’}_{ik}-{\bar{x}’}_{k}\right)}^{2}}},$$

In the formula, ${x’}_{ij}$ and ${x’}_{ik}$ denote the values of the i-th sample in the j-th and k-th evaluation domains, respectively. The mean values of the j-th and k-th evaluation domains, designated as ${\bar{x}’}_{j}$ and ${\bar{x}’}_{k}$, respectively, are calculated as the mean of the observed values. The total number of samples is denoted by *n*.

➃ Calculate conflict $R_{j}$: For each evaluation domain *j*, calculate its conflict with other evaluation domains using Formula (27).
  (27) $$R_{j}=\sum_{k=1}^{n}\left(1-\left|r_{jk}\right|\right),$$
➄ Calculate the information content $C_{j}$: The information content is the product of the standard deviation and the conflict, calculated using Formula (28).
  (28) $$C_{j}=\sigma_{j}\times R_{j},$$
➅ Normalize weights: Normalize the information quantity as shown in Formula (29) to obtain the weights $\omega_{j}$ of each evaluation domain.
  (29) $$\omega_{j}=\frac{C_{j}}{\sum C_{k}},$$

Within the formula, $C_{j}$ denotes the amount of information in the current evaluation domain and $\sum C_{k}$ signifies the sum of the amounts of information in all evaluation domains.

The sixth step of the process is to calculate the mean value of each evaluation area in all samples. The weights of each evaluation area must then be combined, and the total score M of the layout evaluation index must be calculated, as shown in Formula (30).

(30) $$M=\sum_{j=1}^{n}{\bar{x}’}_{j}\omega_{j},$$

In the formula, ${\bar{x}’}_{j}$ denotes the mean value of the jth evaluation domain, while $\omega_{j}$ signifies the weight of the j-th evaluation domain. The quantity n represents the total number of evaluation domains. It can be concluded that the lower the total score M of the layout evaluation indicators, the better the current comprehensive layout indicators.

### 2.5. Experimental Design

#### 2.5.1. Introduction to the Experimental Vehicle

**Figure 8:**
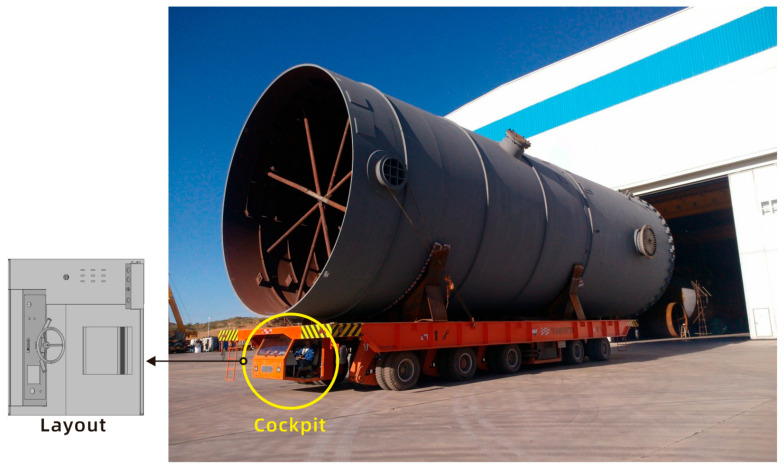
A model of a flatbed transport vehicle.

The present study employed an authentic project as a case study to verify the feasibility of the proposed hybrid optical motion capture and simulation-based quantitative comprehensive evaluation method. Specifically, an evaluation was conducted of the cockpit layout of a flatbed transport vehicle designed specifically for port transportation (see Figure 8; due to confidentiality agreements, the specific vehicle model has been blurred).

The flatbed transport truck under consideration has a maximum load capacity of 200 tons and is fabricated from high-strength steel, thereby ensuring a robust body structure. It is utilized in a broad range of applications, encompassing domains such as construction, mining, logistics, and numerous other industries, where it is employed to address the transportation requirements in a myriad of scenarios.

#### 2.5.2. Selection of Participants

A total of 30 participants were recruited for this experiment (mean age = 28.9, SD = ± 1.37; all were adult males, consistent with the actual circumstances of this occupation). In order to ensure consistency with the simulation parameters that would be employed in subsequent stages, the dimensions of all participants’ body parts and weight parameters were essentially consistent with those of the selected human body model parameters. All participants were in good physical condition, with no significant musculoskeletal disorders, and were capable of independently completing interactive tasks within the cockpit of a specially designed vehicle. Prior to the commencement of the experiment, all participants were required to complete a personal information form and sign an informed consent form.

#### 2.5.3. Experimental Setup and Procedure

The experiment was conducted within a manufacturing facility located at the flatbed transport site. To ensure relatively constant daylight conditions, the experiment was conducted under the illumination of a fixed light source. As illustrated in Figure 8, the experimental vehicle’s cabin is characterized by dimensions of 2.2 m × 1.5 m × 1.2 m, accommodating a single occupant. The vehicle driving module is located in front of the seat, while the special equipment operation module is located on the right side of the seat.

**Figure 9:**
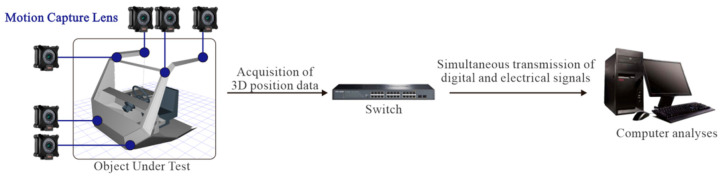
Optical motion capture equipment description.

The NOKOV Optical 3D Motion Capture System is composed of six 850 nm infrared cameras installed within the cockpit (see Figure 9), with data collected at a rate of 90 Hz. A total of 53 reflective markers are placed on the participant’s body, in accordance with the modified Helen Hayes marker set protocol. The markers are fixed to key anatomical landmarks on the head, trunk, pelvis, and upper and lower limbs (including the acromion, lateral epicondyle of the elbow, ulna and radius styloid processes, iliac crest, lateral condyle of the femur, and lateral malleolus) in order to track the movement of each body segment with maximum accuracy. Prior to the collection of data, the T-shaped calibration rod is utilized to calibrate the capture space formed by the six cameras, thereby defining the global coordinate system and minimizing measurement errors. The fundamental principle underlying the identification of marker points is to ascertain their precise spatial locations by employing multiple infrared cameras to locate the marker points. The LED lights on the optical motion capture camera panel emit infrared light of a specific wavelength, which is reflected off the reflective markers on the object being captured. The markers are coated with a reflective material that functions by reflecting infrared light back to the camera. Subsequently, the reflected infrared light undergoes signal processing, wherein an FPGA captures the image and performs algorithmic processing to obtain the two-dimensional coordinates of the reflective markers within the camera. A motion capture system generally comprises multiple motion capture lenses. The calibration of these lenses is performed to ascertain their precise positions relative to one another, thereby yielding three-dimensional coordinates. Subsequent to data collection, the 3D trajectories of each marker point are reconstructed and annotated using XINGYING software (v. 3.3.1). The process generates 3D coordinates, quaternions, and Euler angles for each body segment, which are then used to construct a digital human model for the subsequent analysis.

Prior to the commencement of the experiment, each participant was acquainted with the simulated experimental environment and the experimental process of the study. This was undertaken to facilitate their familiarization with the experimental environment and process. During the experiment, the experimenter selected a simulated work posture and issued operational commands, and the participants were required to complete the corresponding work tasks. Subsequent to the completion of each work posture, participants were permitted a period of rest lasting 60 s. This process was then repeated until all work postures had been recorded. The installation points of the optical motion capture equipment and the data transmission method are illustrated in Figure 9, while the work posture images to be recorded are delineated in Table 14.

Data synchronization: In order to ensure precise temporal alignment between the kinematic and EMG data, a hardware synchronization method was employed. The NOKOV motion capture system functioned as the primary device. The transmission of a transistor–transistor logic (TTL) pulse from the motion capture controller to the external trigger input of the EMG acquisition system was achieved by means of a synchronization cable. This signal initiated and terminated data recording for both systems concurrently, thereby ensuring the synchronization of the motion capture and EMG data streams.

### 2.6. Statistical Analysis

The statistical analysis in this study was conducted using IBM SPSS Statistics 26.0 software, and all quantitative data are presented as the mean ± standard deviation (mean ± SD). In order to ascertain any disparities in evaluation scores between the ‘original design’ and ‘optimized design’ layout schemes, paired-sample t-tests were conducted for each of the nine evaluation indicators. The test method was deemed suitable for the within-subject design of the study (*n* = 30), in which each participant was exposed to both experimental conditions.

The Shapiro–Wilk test confirmed that the paired difference scores for all nine evaluation indicators were normally distributed (all *p* > 0.05), thereby satisfying the prerequisites for t-tests. To control the cumulative risk of type I errors resulting from nine independent tests on nine assessment indicators ($M_{\left(A1\right)}$, $M_{\left(A2\right)}$, $M_{\left(B1\right)}$, $M_{\left(B2\right)}$, $M_{\left(C1\right)}$, $M_{\left(C2\right)}$, $M_{\left(C3\right)}$, and $M_{\left(C4\right)}$, and the total score, M), the Bonferroni correction was applied, adjusting the significance level α from 0.05 to the corrected α′ = 0.05/9 ≈ 0.0056.

## 3. Results

### 3.1. Experimental Results

#### 3.1.1. Evaluation Results

The experimental results indicate that the human skeleton identified by the optical lens in different postures is basically intact, with no obvious defects or missing joints, and the recognition effect is good. The collection of work posture data was undertaken, resulting in the acquisition of 30 sets of human kinematic data for a range of interactive tasks. The participants’ body dimensions were utilized to select the P5, P50, and P95 male parameters as the parameter settings for the virtual digital human. The human kinematic data obtained were imported into JACK for the purpose of a simulation analysis under various work postures. The layout evaluation score of the original scheme was calculated using an evaluation scoring program generated by the layout evaluation mapping model and formula, with the results shown in Table 15.

A subsequent analysis of the assessment results revealed the following issues with the cab of the flatbed transport vehicle:

① It is evident that the current H-point position is suboptimal, resulting in the steering wheel, center console, pedals, and other components being beyond the reach of the passenger, thereby compromising both passenger comfort and well-being, and potentially inducing fatigue.
② The location of the emergency stop button, which is positioned outside the maximum reachability range of the passenger’s hand, and the red button’s lack of visibility from the driver’s perspective, pose a significant safety hazard.
③ The positioning of the micro-electric control panel on the right side of the vehicle is suboptimal, with the joystick and some buttons being located beyond the maximum reachable range of the operator’s hand. This design choice results in reduced operator comfort and increased fatigue when operating the joystick.
④ The positioning of the air conditioning unit is suboptimal, with the control buttons being located at a distance that exceeds the maximum reachable range of the passenger’s hands. This necessitates a significant alteration in the passenger’s posture to adjust the air conditioning, thereby compromising both the comfort of use and the passenger’s physical well-being.
⑤ The configuration of the center console could be improved through further refinement, as certain button and indicator light colors currently present a challenge to the driver when in a standard driving position. The positioning of monitoring displays and indicator lights on the left, center, and right sides of the center console has a certain impact on the driver’s ability to monitor the vehicle and equipment status efficiently.

#### 3.1.2. Optimization of Human–Machine Layout Based on Evaluation Results

In response to the prevailing human–machine layout issues in the cab of flatbed transport vehicles, the following optimization proposals are hereby presented.

**Figure 10:**
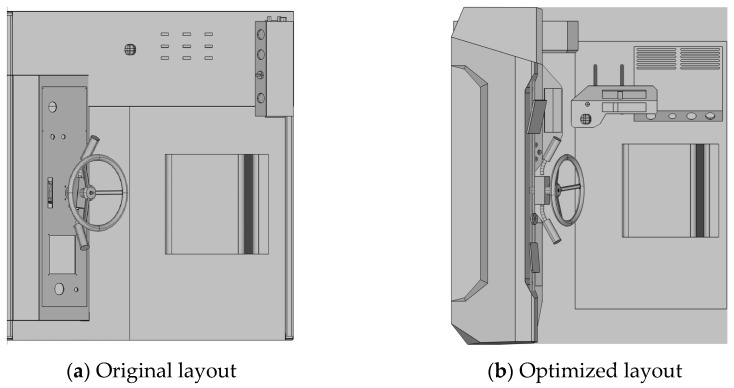
Comparison of layout schemes before and after optimization.

The initial step in the process is the optimization of the H-point position, followed by the redesign of the interior layout of the cab. The second stage of the process is to reorganize the functional zones of the micro-electronic panel. The third step in the process is the relocation of the air conditioning system. The fourth point to consider is the redesign of the central control layout, with the objective of integrating functional zones. The finalized optimized layout is displayed in Figure 10, in comparison to the original layout.

### 3.2. Verification

The present study proposes a quantifiable, comprehensive evaluation method that combines optical motion capture and simulation processes. The method was applied to evaluate the original layout design of the flatbed truck cab, the layout was optimized based on the evaluation results, and the flatbed truck cab optimization design project was ultimately completed. In order to validate the effectiveness of the proposed evaluation method and the design scheme derived from it, a cross-validation was conducted by comparing the comprehensive evaluation scores and electromyographic signals under typical tasks.

#### 3.2.1. Re-Evaluate

**Figure 11:**
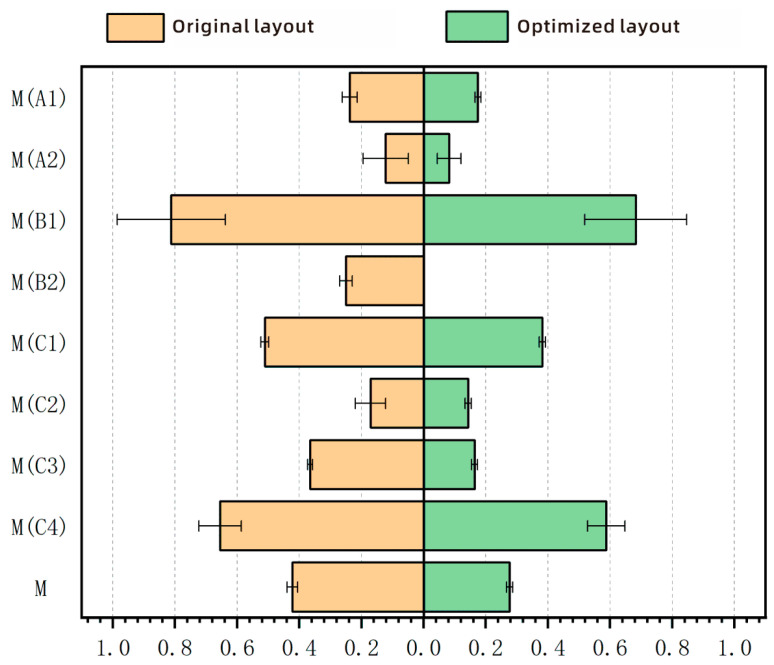
Optimization before and after layout evaluation scores.

The establishment of an optimized simulation model of the special vehicle cab was undertaken, with a simulation analysis conducted concurrently whilst maintaining other conditions as constant. The simulation results were then calculated using a comprehensive evaluation model. As illustrated in Figure 11 and outlined in Table 16, the layout evaluation index scores for the two schemes are demonstrated.

The results of the paired-sample t-test indicate that the optimized layout shows statistically significant improvements. As shown in Table 17, the total evaluation score for the optimized layout (0.277 ± 0.010) is significantly lower than that for the original layout (0.422 ± 0.017). The analysis results show t(29) = 25.8, *p* < 0.001, and after applying the Bonferroni correction, the *p*-value remained highly statistically significant (*p* < 0.0056). Furthermore, an in-depth analysis of specific ergonomic dimensions revealed significant improvements in the local design. Notably, the lower limb accessibility was greatly enhanced, with the assessment score decreasing from 0.25 ± 0.020 to 0.00, reaching the optimal theoretical level. Additionally, the trunk posture rationality also showed significant improvement, with the score decreasing from 0.171 ± 0.049 to 0.143 ± 0.017. These improvements in the two key sub-indicators also passed the Bonferroni correction test. This indicates that the optimized design is a global improvement that successfully addresses the original design’s specific shortcomings in terms of lower limb operation and trunk load. These findings suggest that optimizing the configuration of devices such as the pedals enables the new layout to better accommodate drivers of different body sizes and reduce their physiological load. In summary, the optimized design is superior to the original in terms of layout and comfort.

#### 3.2.2. Cross-Validation Based on EMG

**Figure 12:**
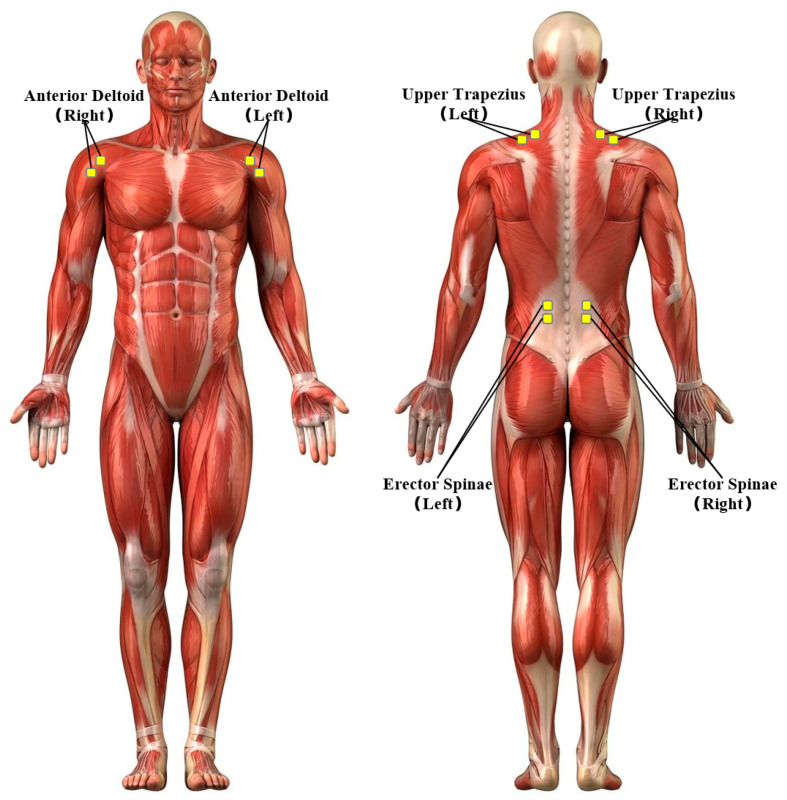
Schematic diagram of electromyography electrode placement.

Electromyography (EMG) is a technique used to record and analyze muscles’ electrical activity. The device utilizes sensors to detect bioelectric signals generated during muscle contraction, thereby providing insights into muscle activation levels, the degree of fatigue, and neural control patterns [22]. The utilization of this method is prevalent in the evaluation of the impact that work postures exert on muscle load. In order to validate the effectiveness of this layout optimization design method for special vehicle cab layout optimization, the experiment involved the attachment of surface EMG electrodes to key muscles in the participants’ neck, shoulder, and waist areas to obtain EMG data before and after cab optimization. The specific muscles monitored were the upper trapezius (UT) for the neck and shoulder load, the anterior deltoid (AD) for upper limb exertion during control manipulation, and the lumbar erector spinae (LES) for lower back postural strain. Prior to electrode attachment, the skin surface was shaved (if necessary) and cleansed with alcohol in order to reduce impedance. The placement of bipolar Ag/AgCl surface electrodes was conducted in accordance with the recommendations of the SENIAM (Surface ElectroMyoGraphy for the Non-Invasive Assessment of Muscles) project, with the objective of ensuring consistency and comparability. As illustrated in Figure 12, the precise locations for electrode placement are indicated.

**Figure 13:**
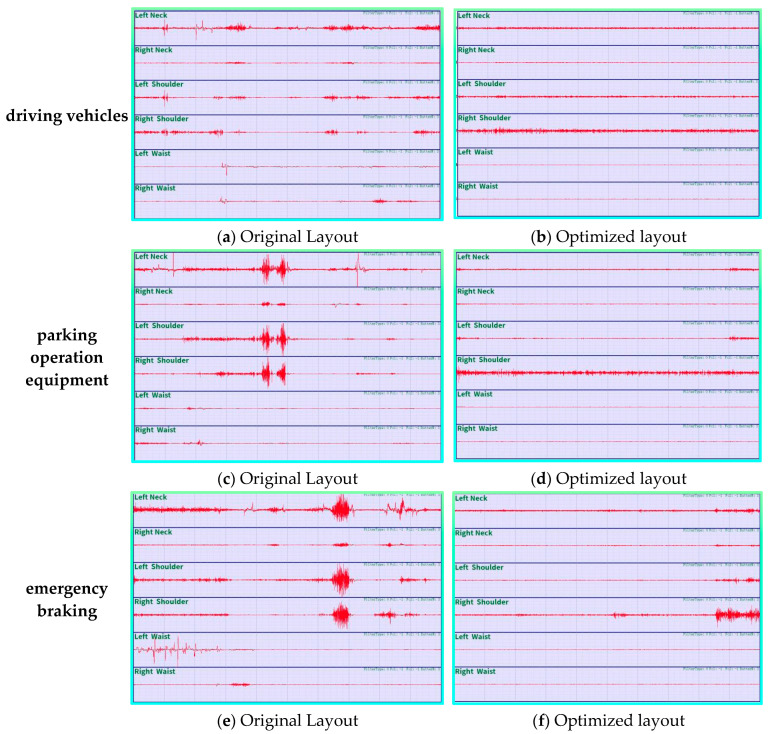
EMG signal diagrams for optimized and non-optimized solutions in different tasks.

Utilizing three archetypal work postures (driving, parking, and emergency braking) as exemplars, Figure 13 elucidates the alterations in muscle electrical signals (mean values) at diverse body locations under the two schemes.

Changes in EMG signals from the neck, shoulders, and waist muscle tissues indicate that under three typical working postures, the original design results in significantly increased and irregular fluctuations in local muscle group EMG signals, with overall muscle activity levels being high and prone to fatigue. Conversely, the optimized design demonstrates relatively stable EMG signals at diverse muscle points, diminished muscle activity levels, and enhanced muscle control. Consequently, the optimized design provides enhanced driving comfort in comparison with the original design.

## 4. Discussion

### 4.1. Key Findings and Interpretation

This study successfully proposed and validated a quantitative, comprehensive evaluation method combining optical motion capture and simulation. The core finding is that the optimized cab layout achieved through this method resulted in a significant reduction of 34.4% in the comprehensive evaluation score (from 0.422 to 0.277), and the neck, shoulder, and waist muscle loads (EMG signals) of the test subjects during typical tasks were also significantly reduced. This finding suggests that the proposed method is not only effective in the model, but also results in significant improvements in actual physiological effects.

### 4.2. Comparison with Existing Research

This study’s core contribution lies in the innovative methodology it employed to effectively address several key challenges currently facing human factors engineering assessment.

Traditional digital human models (DHMs) have been the focus of academic attention in terms of improving the accuracy of simulation data sources due to the limitations of their pose databases or reliance on manual positioning [23]. This results in discrepancies between their poses and real-world dynamic operations. These discrepancies lead directly to inaccuracies in the reachable and visible domains and in biomechanical analyses—a problem that has been widely discussed. Research has shown that the accuracy of standard DHMs significantly decreases when predicting work postures for non-standard or dynamic tasks, potentially leading to an underestimation or overestimation of the operator’s physiological load [24]. This study introduces an optical motion capture system that collects three-dimensional kinematic data directly from real operators to drive the DHM. This ensures the ecological validity of the assessed postures and aligns with recent research trends that emphasize using real-world data to validate or drive DHMs to enhance their reliability in human–machine interaction assessments [25]. Unlike studies that only use motion capture data for static posture validation in DHMs, this study used the dynamic capture data stream directly as the input for the simulation analysis, achieving a transition from ‘static validation’ to ‘dynamic driving’.

In terms of the comprehensiveness and quantification of the evaluation methods, the existing studies typically employed multiple human factors assessment tools (e.g., RULA, OWAS, and LBA methods) for independent analyses when applying simulation software (e.g., JACK and DELMIA) [26]. However, these tools operate independently and often produce discrete risk levels or local scores. Integrating this fragmented information to inform global layout decisions poses a long-standing challenge, known as the ‘information silo’ problem, and academic circles have recognized the limitations of single assessment tools. Consequently, they have begun to explore frameworks for multi-criteria integrated assessments [27]. This paper proposes a comprehensive, quantitative evaluation model that standardizes multi-source data (field of view, reachable area, RULA, OWAS, etc.) and uses the CRITIC objective weighting method to dynamically integrate it. This ultimately produces a single, comparable comprehensive score, addressing the fragmentation of evaluation results and providing an objective, unified quantitative basis for the horizontal comparison and selection of design schemes. This represents an important supplement to and deepening of existing evaluation methodologies.

In terms of the physiological validation of the research findings, this study used EMG experiments to confirm that the optimized scheme significantly reduces the muscle load in the neck, shoulders, and lower back. This finding is highly consistent with the conclusions of the extensive literature on the effects of ergonomic interventions. Numerous studies, for example, have demonstrated that optimizing the seat parameters, control panel layout, or tool design can effectively reduce muscle fatigue and the risk of work-related musculoskeletal disorders (WMSDs) among operators [28]. The EMG data from this study provide direct physiological evidence for the effectiveness of layout optimization and reaffirm that the ‘optimal’ solution predicted by the comprehensive evaluation model can translate into tangible physiological benefits for operators. This creates a closed-loop process of ‘model prediction–design intervention–physiological validation’, thereby enhancing the persuasiveness of the entire methodology.

### 4.3. Research Significance and Application Prospects

The most significant theoretical contribution of this study lies in providing a paradigm shift from ‘experience-based judgement’ to ‘data-driven global optimization’ for the layout design of complex human–machine interfaces. This method is especially pertinent for highly customized industries, such as the production of specialized vehicles, as it facilitates the low-cost, high-efficiency iterative optimization of multiple designs without the necessity for physical prototypes. This effectively circumvents the high costs typically associated with traditional experimental methods. The core framework of this method combines motion capture, simulation, and comprehensive evaluation processes, thereby demonstrating high transferability and holding promise for future applications in the design and evaluation of other complex human–machine systems, including aircraft cockpits, precision instrument control panels, and construction machinery cabins.

### 4.4. Limitations and Future Research Directions

Despite the favorable outcomes of this study, its inherent limitations offer direction for future research. The simulation did not take into account the impact of real-world environmental factors, including but not limited to lighting, vibration, and noise, on driver comfort. Future research could explore integrating this model with virtual reality (VR) technology to provide an immersive visual experience while simulating various environmental disturbances, thereby rendering the assessment more comprehensive. The motion capture scheme in this study primarily focused on the main skeletal structure of the body and did not include detailed finger movements. The subsequent phase of research will involve the integration of data gloves or high-precision finger joint recognition technology to enhance the evaluation of interface layouts necessitating meticulous operations, such as control panels. The evaluation tasks in this study were predefined. Subsequent research endeavors will encompass the design and exploration of increasingly complex, non-predefined emergency task scenarios. These scenarios will be utilized to assess the robustness of the layout when subjected to high-pressure conditions.

Another important methodological limitation of this study was the manner in which muscle loads were evaluated. We primarily used mean electromyography (EMG) amplitudes to compare muscle activity levels under different conditions, inferring improvements in comfort based on this. While this indicator effectively reflects overall muscle activation, it is not a direct or robust indicator of muscle fatigue. A more rigorous assessment of local muscle fatigue would require an analysis of changes in EMG signal spectra. As demonstrated by Hyeonseok Kim in his study, during sustained muscle contraction, the median frequency (MDF) or average frequency (MNF) of the EMG signal power spectrum gradually shifts towards the low-frequency region [29]. The decrease in this value is an internationally recognized, reliable indicator of muscle fatigue. Therefore, the conclusion of this study should be interpreted as ‘the optimized scheme reduced muscle activation levels’, rather than proving that it ‘slowed down the rate of fatigue onset’. Future research must incorporate spectral analyses into the assessment system to evaluate the true impact of different cab layouts on operator muscle fatigue more directly and quantitatively by tracking changes in MDF and MNF.

The comprehensive evaluation model in this study currently relies on ‘crisp’ logic and thresholds to quantify various indicators. Nevertheless, this approach is problematic for many people because engineering standards are inherently vague (e.g., the boundary between ‘comfortable’ and ‘slightly uncomfortable’). To better address this inherent uncertainty, future research should consider incorporating fuzzy logic to optimize the evaluation framework. Specifically, it is possible to fuzzify the outputs of each module, i.e., to describe them using membership functions that indicate the degree to which a state belongs to different fuzzy sets (e.g., the resulting data can be categorized as either comfortable, moderate, or poor). Subsequently, a fuzzy inference system (FIS) based on expert human factor knowledge can be constructed to integrate fuzzy information from different modules through a series of ‘IF–THEN’ rules, thereby arriving at a comprehensive judgement on the overall layout quality. Expert systems based on fuzzy logic can effectively integrate multi-source, imprecise information to perform risk assessments that align more closely with human expert judgment logic [30]. The integration of this methodology will serve to enhance the maturity and robustness of our model in addressing uncertainties in evaluation criteria.

The present study concentrated chiefly on verifying the simulation results through macro-level evaluation scores of the final design scheme and direct physiological effects (EMG), without conducting a more in-depth biomechanical validation of the physical plausibility of the simulated postures themselves. It is recommended that future research incorporates inverse dynamics analysis. Specifically, the kinematic data obtained from motion capture can be used as inputs to a detailed rigid body biomechanical model to solve the joint torques and reaction forces required to produce these movements. This method involves a twofold approach: firstly, it verifies whether the captured postures are physiologically feasible (e.g., whether the torques are within a reasonable range); secondly, it provides more accurate and quantitative joint load data than traditional observation assessment tools, thereby enabling a more in-depth comparison of the designs. Analyses based on inverse dynamics can reveal potential injury risks that cannot be observed using traditional methods [31]. Furthermore, the integration of motion control theories, such as energy optimization (energy-based), can serve to verify the compliance of the observed operational postures with fundamental human movement principles, including ‘minimum energy expenditure’. This process of validation serves to further substantiate the naturalness of the simulated behavior.

The present research framework is chiefly concerned with comfort and rationality at the biomechanical level, with no direct incorporation of the driver’s cognitive and psychophysiological states (e.g., stress and fatigue) into the optimization closed loop. Future research should aim to expand layout optimization processes into an adaptive system that incorporates physiological feedback. The initial step in this methodology is the integration of multimodal biosignal sensors, such as electroencephalogram (EEG), for the monitoring of cognitive load and drowsiness, and a heart rate variability (HRV) analysis for the assessment of psychological stress. The fusion and analysis of these multimodal signals using artificial intelligence technology constitutes the core of achieving precise driver state detection [32]. Consequently, future evaluation systems have the potential to incorporate an artificial intelligence-powered ‘physiological state prediction module.’ During the virtual simulation phase, the module has the capacity to predict the cognitive load and fatigue levels that specific schemes may induce based on task processes and layout design. These predictions can then be incorporated as a new key indicator into the comprehensive evaluation score.

The evaluation framework of this study focuses exclusively on the ergonomic performance of operators, overlooking the potential impact of human–machine interaction on the material fatigue of physical components. It is hypothesized that a more comfortable operating posture for humans may alter their force application patterns, thereby inducing new, unexpected stress cycles on metal or polymer components such as joysticks and seats. This, in turn, could affect their mechanical reliability and service life. It is recommended that future research focus on establishing a ‘human–machine’ joint simulation and collaborative optimization framework. This framework utilizes biomechanical analyses (e.g., inverse dynamics) to calculate the dynamic loads exerted by the human body on machine components. These loads are then used as inputs for a finite element analysis (FEA) and fatigue life prediction of the components. The aim of this approach is to achieve the dual optimization of human factors (comfort, fatigue) and machine performance (structural strength, lifespan). This is intended to result in a more comprehensive and reliable system-level optimal design.

### 4.5. Relationship with Emerging Intelligent Monitoring Systems and Future Integration

As demonstrated by recent studies utilizing smart electronic devices to monitor SAR (specific absorption rate) and temperature changes in human tissue [33], the adaptive real-time measurement of dynamic ergonomic environments represents a cutting-edge direction in this field. The two methodologies under discussion have different positionings and advantages. The primary benefits of wearable intelligent systems are attributable to their capacity for real-time monitoring and environmental adaptability [34]. The system has the capacity to continuously capture physiological and kinematic data of operators in real, unpredictable working environments, thereby enabling real-time feedback and personalized interventions. This is of crucial importance in the field of operator condition monitoring. In contrast, the ‘motion capture + simulation’ method proposed in this study functions as an offline predictive tool for design and evaluation. Its primary strengths lie not in real-time monitoring but in its predictive design capabilities and comprehensive evaluation. It facilitates comprehensive, methodical biomechanical and environmental interaction assessments of numerous design options prior to physicalization, thereby addressing design flaws at the source. However, these two methods are not mutually exclusive but rather highly complementary, collectively pointing towards the future of human factors engineering—digital twins. The optimal framework for the future would be the integration of the advantages of both approaches. Utilizing the methods developed in this study, a high-fidelity ‘operator–cockpit’ digital twin model can be constructed and validated through a single high-precision optical motion capture and biomechanical simulation. Subsequently, during routine utilization, operators are required to wear lightweight sensors that feed real-time, sparse data streams into the aforementioned calibrated digital twin model. The model is then able to use these real-time inputs, combined with its integrated complete biomechanics and environmental constraints, to calculate comprehensive ergonomic assessment results in real time. This integration will facilitate comprehensive, real-time, and adaptive human factor engineering assessments in actual dynamic tasks, thereby representing a paradigm shift from ‘offline assessments’ and ‘simple monitoring’ to ‘real-time comprehensive analyses’. Moreover, this development signifies that conventional assessment models such as the RULA, OWAS, and CA can be transformed into a dynamic, real-time assessment interface. The crux of this objective is to substitute conventional manual observations with automated real-time posture capture technology. Once the system can continuously obtain the operator’s real-time joint angles, it can run the scoring logic of the RULA or OWAS in real time and continuously through a background computing engine. Furthermore, by introducing fuzzy logic to smooth out scoring thresholds, the system can avoid abrupt changes in evaluation results at critical points. It is evident that these real-time, dynamically changing evaluation scores can be displayed through ambient lighting in the cockpit or haptic feedback from the seat. This non-intrusive method of alerting the operator to adjust their uncomfortable posture is a key feature of the system. This configuration establishes a closed-loop, adaptive ‘posture–evaluation–feedback’ system.

The key to realizing this vision lies in introducing AI technology as an intelligent engine for interpreting real-time data. The capacity of deep learning frameworks for the advanced interpretation of real-time motion data, rather than merely recording it, is a significant development [35]. In the future, the deployment of an AI model pre-trained with extensive simulation data in a digital twin system is envisioned. This model has the capacity to analyze sparse motion signals from wearable IMU sensors in real time, thereby enabling the instant prediction of changes in the operator’s complete posture, motion intent, and even injury risk trends. This will enable the digital twin to function not merely as a passive virtual mirror but as an intelligent entity with predictive and decision-support capabilities.

## 5. Conclusions

Specialized vehicles represent indispensable components within the modern engineering, military, and firefighting domains, characterized by highly customized human–machine layouts in their cockpits. It is evident that traditional evaluation methods are rife with three major drawbacks. Firstly, experimental methods rely on physical prototypes, resulting in high costs and protracted cycles. Secondly, mathematical modelling tends to become entrenched in local optima, thereby ignoring the need for overall layout coordination. Thirdly, simulation tools are characterized by fragmented functions, with evaluation results lacking systematic quantitative standards. The aforementioned issues result in subjective and unreliable layout designs, which have a detrimental effect on driver comfort and safety. The present paper proposes a hybrid optical motion capture and simulation-based quantitative comprehensive evaluation method. This optimized design method employs optical motion capture information as the source of human kinematic data. The method utilizes an optical motion capture system to accurately obtain three-dimensional kinematic data during driver operations, thereby generating a digital model of real-world postures. The approach employs a multi-method fusion assessment, integrating six assessment methods, including a visual analysis, reachability mapping, RULA, and OWAS, to construct a comprehensive indicator system encompassing eight major areas. The system has been developed to convert non-quantitative data into ratio scores, to quantify data through normalization and unified dimensions, and to use the CRITIC weighting method to dynamically allocate weights to each area. In practical applications, this provides tools for the layout design of special vehicle cockpits, proposing more reasonable layout optimization designs that significantly enhance operator comfort (EMG validated), safety (reduced blind spots), and efficiency (optimized control accessibility).

The Integration of finger joint posture recognition in future research is intended to achieve several objectives. These objectives include the expansion of fine motion recognition, the enhancement of the accuracy of hand operation assessments, and the improvement of the accuracy and comprehensiveness of this optimization design method. In addition, the investigation will encompass the consideration of further influential factors, including the integration of environmental interference coefficients on comfort, the development of an automated assessment platform, and the realization of real-time layout optimization feedback. In future work, the objective is to validate the optimization design method in a broader range of practical application scenarios, improve its rationality and universality in the human–machine layout optimisation of special vehicle cockpits, and continuously optimize and improve the model during validation. This will enable more special vehicle operators to benefit from the promotion of this optimization design method.

## Figures and Tables

**Table 1:** Head rotation angle range.

Rotating Dimension	Horizontal	Vertical
maximum range	−90°~90°	−70°~50°
comfort zone	−60°~60°	−30°~30°

**Table 2:** Range of motion of the shoulder joint and elbow joint.

Symbol	Direction of Movement	Physiological Limit Angle	Comfort Reachability Value Angle
$\theta_{1}$	flexion	0°~180°	0°~120°
	extension	0°~60°	0°~20°
$\phi_{1}$	Abduction	0°~180°	0°~90°
	Adduction	0°~20°	0°~10°
$\theta_{2}$	Flexion or extension	0°~150°	30°~130°
$\phi_{2}$	pronation	0°~80°	0°~60°
	supination	0°~90°	0°~60°

**Table 3:** Range of motion of the trunk.

Symbol	Direction of Movement	Physiological Limit Angle	Maximum Reachability Value Angle
$\theta_{3}$	Trunk flexion	0°~60°	0°~30°
$\phi_{3}$	Trunk lateral bending	0°~30°	0°~15°

**Table 4:** Reachability boundary formula.

Boundary	Formula
outer boundary ($\theta_{k}=0$)	$\left\{\begin{array}{c} x_{f}=\left(L_{thigh}+L_{shank}\right)\sin\theta_{h}\\ y_{f}=\left(L_{thigh}+L_{shank}\right)\cos\theta_{h} \end{array}\right.$ $\theta_{h}\in \left[\theta_{h\_min},\;\;\theta_{h\_max}\right]$
inner boundary ($\theta_{k}=\theta_{k\_max}$)	$\left\{\begin{array}{c} x_{f}=L_{thigh}\sin\theta_{h}-L_{shank}\sin{(\theta }_{h}+\theta_{k\_max})\\ y_{f}=L_{thigh}\cos\theta_{h}-L_{shank}\cos{(\theta }_{h}+\theta_{k\_max}) \end{array}\right.$ $\theta_{h}\in \left[\theta_{h\_min},\;\;\theta_{h\_max}\right]$
left boundary ($\theta_{h}=\theta_{h\_min}$)	$\left\{\begin{array}{c} x_{f}=L_{thigh}\sin\theta_{h\_min}-L_{shank}\sin{(\theta }_{h\_min}+\theta_{k})\\ y_{f}=L_{thigh}\cos\theta_{h\_min}-L_{shank}\cos{(\theta }_{h\_min}+\theta_{k}) \end{array}\right.$ $\theta_{k}\in \left[0,\;\;\theta_{k\_max}\right]$
right boundary ($\theta_{h}=\theta_{h\_max}$)	$\left\{\begin{array}{c} x_{f}=L_{thigh}\sin\theta_{h\_max}-L_{shank}\sin{(\theta }_{h\_max}+\theta_{k})\\ y_{f}=L_{thigh}\cos\theta_{h\_max}-L_{shank}\cos{(\theta }_{h\_max}+\theta_{k}) \end{array}\right.$ $\theta_{k}\in \left[0,\;\;\theta_{k\_max}\right]$

**Table 5:** RULA posture rating rules.

Part	Score and Corresponding Posture			
Upper arm (Group A)	Score 1: Natural droop	Score 2: Angle with trunk ≤ 20°	Score 3: Angle with trunk 20°~45°	Score 4: Angle with trunk > 45° or hyperextension
Forearm (Group A)	Score 1: Angle with upper arm 60°~100°	Score 2: Angle with upper arm < 60°	Score 2: Angle with upper arm > 100°	—
Wrist	Score 1: Neutral position	Score 2: flexion ≥ 15°	Score +1: Wrist twist	—
Neck (Group B)	Score 1: Neutral position	Score 2: Forward flexion > 10° or lateral flexion/twisting	—	—
Torso (Group B)	Score 1: Standing upright	Score 2: Forward flexion > 10° or lateral flexion/twisting	—	—

**Table 6:** Load and muscle use rating scale.

Load Source	Scoring Rules	Score
External load	No weight	+0
	Weight ≤ 2 kg	+1
	Weight > 2 kg	+2
Muscle use	Static posture < 1 min	+0
	Static ≥ 1 min or repeated > 4 times/minute	+1

**Table 7:** RULA total score adjustment.

Posture Score	Load Score	Muscle Usage Score	Extra Points (Δ*e*)
1~3	0	0	+0
1~3	1~2	0~1	+1
4~6	0	0	+1
4~6	1~3	0~1	+2
≥7	Any	Any	+3

**Table 8:** RULA assessment scores and fatigue risk rating.

$P_{RULA}$	Risk Level
1~2	Level 1
3~4	Level 2
5~6	Level 3
≥7	Level 4

**Table 9:** OWAS posture rating rules.

Part	Posture Coding and Scoring			
Back	Score 1: Standing upright or slightly flexion	Score 2: Forward flexion ≤ 45°	Score 3: Severe forward flexion > 45°	Score 4: Twisting or flexion sideways
Arms	Score 1: Hands below shoulder level	Score 2: Single arm or both arms above shoulder level	Score 3: Single or double arm forward extension	—
Legs	Score 1: Sitting posture	Score 2: Standing or walking	Score 3: Single leg flexion	Score 4: Both legs in flexion
Load	Score 1: Weight ≤ 10 kg	Score 2: Weight 10~20 kg	Score 3: Weight > 20 kg	—

**Table 10:** OWAS fatigue risk classification table.

Back	Arms	Legs	Load	Fatigue Risk Level
1	1	1	1	Level 1
2	1	2	2	Level 2
2	2	3	1	Level 3
3	2/3	4	3	Level 4
4	Any	Any	2/3	Level 4

**Table 11:** Range of joint angle evaluation.

Joint Mobility Trends	Neutral Angle	Comfort Angle Range	High-Risk Angle Threshold
Shoulder abduction	0°	0°~20°	>30°
Elbow flexion	0°	80°~120°	<60° or >140°
lumbar flexion	0°	0°~20°	>30°
Knee flexion	0°	90°~110°	<70° or >130°

**Table 12:** Deviation description and rating.

Deviation	Risk Level	Rating
All joints are close to neutral position.	high	≤2
Moderate deviation in some joints	medium	3~5
Severe misalignment of multiple joints	low	>5

**Table 13:** Scoring formula for each evaluation module.

Evaluation Module	Scoring Formula
$M_{\left(A1\right)}$	$M_{\left(A1\right)}=\left(P_{1}+P_{2}\right)/2$
$M_{\left(A2\right)}$	$M_{\left(A2\right)}=P_{3}$
$M_{\left(B1\right)}$	$M_{\left(B1\right)}=\left(P_{4}+P_{5}\right)/2$
$M_{\left(B2\right)}$	$M_{\left(B2\right)}=\left(P_{6}+P_{7}\right)/2$
$M_{\left(C1\right)}$	$M_{\left(C1\right)}=\left(P_{8}+P_{10}+P_{11}\right)/3$
$M_{\left(C2\right)}$	$M_{\left(C2\right)}=\left(P_{9}+P_{11}\right)/2$
$M_{\left(C3\right)}$	$M_{\left(C3\right)}=\left(P_{8}+P_{10}+P_{11}\right)/3$
$M_{\left(C4\right)}$	$M_{\left(C4\right)}=\left(P_{8}+P_{10}+P_{11}\right)/3$

**Table 14:** Work postures involved in the experiment.

Task Name	Task Description	Controls Involved
Driving vehicles	Drive the vehicle to the destination, completing tasks such as turning, accelerating, and decelerating along the way.	Start button, circuit control panel, steering wheel, pedals
Parking operation equipment	Control the vehicle platform to lift, lower, or rotate to a certain angle.	Right joystick, low-voltage control panel
Emergency braking	Immediately stop all tasks, stop the vehicle, and turn off the power.	Foot pedal, emergency stop button, circuit control panel

**Table 15:** Original layout evaluation score.

Layout	$M_{\left(A1\right)}$	$M_{\left(A2\right)}$	$M_{\left(B1\right)}$	$M_{\left(B2\right)}$	$M_{\left(C1\right)}$	$M_{\left(C2\right)}$	$M_{\left(C3\right)}$	$M_{\left(C4\right)}$	M
Original Layout	0.238 ± 0.024	0.122 ± 0.073	0.812 ± 0.174	0.25 ± 0.020	0.511 ± 0.013	0.171 ± 0.049	0.365 ± 0.008	0.655 ± 0.068	0.422 ± 0.017

**Table 16:** Optimization before and after plan scoring.

Layout	$M_{\left(A1\right)}$	$M_{\left(A2\right)}$	$M_{\left(B1\right)}$	$M_{\left(B2\right)}$	$M_{\left(C1\right)}$	$M_{\left(C2\right)}$	$M_{\left(C3\right)}$	$M_{\left(C4\right)}$	M
Original Layout	0.238 ± 0.024	0.122 ± 0.073	0.812 ± 0.174	0.25 ± 0.020	0.511 ± 0.013	0.171 ± 0.049	0.365 ± 0.008	0.655 ± 0.068	0.422 ± 0.017
Optimized layout	0.175 ± 0.010	0.082 ± 0.038	0.683 ± 0.164	0 ± 0	0.382 ± 0.010	0.143 ± 0.017	0.164 ± 0.012	0.588 ± 0.068	0.277 ± 0.010

**Table 17:** Results of *t*-tests for paired samples of key evaluation indicators for layout optimization schemes before and after optimization (*n* = 30).

Assessment Indicators	Original Layout	Optimized Layout	t	df	*p*	Is it Significant After Bonferroni Correction?
M	0.422 ± 0.017	0.277 ± 0.010	25.8	29	<0.001	Yes
$M_{\left(B2\right)}$	0.25 ± 0.020	0 ± 0	16.4	29	<0.001	Yes
$M_{\left(C2\right)}$	0.171 ± 0.049	0.143 ± 0.017	19.3	29	<0.001	Yes

## Data Availability

The data that support the findings of this study are available from the corresponding author, Qingbin Wang, upon reasonable request.

## Abbreviations

The following abbreviations are used in this manuscript:

RULA	Rapid Upper Limb Assessment
OWAS	Ovako Working Posture Analysis System
EMG	Electromyographic
VA	Visual Analysis
RA	Reachability Analysis
LBA	Lower Back Analysis
CA	Comfort Assessment
